# Rats exposed to a low resource environment in early life display sex differences in blood pressure and pro-inflammatory markers in the brain and kidney during adulthood

**DOI:** 10.1186/s13293-026-00842-8

**Published:** 2026-01-30

**Authors:** Jonna Smith, Savanna Smith, Kylie Jones, Angie Castillo, Jessica L. Bolton, Ahfiya Howard, Luis Colon-Perez, Faith Femi-Ogunyemi, Allison Burkes, Mark Cunningham

**Affiliations:** 1 Department of Physiology and Anatomy, University of North Texas Health Science Center, Fort Worth, TX USA; 2 Neuroscience Institute, Georgia State University, Atlanta, GA USA; 3 School of Social Work, East Texas A&M University, Commerce, TX USA; 4 Department of Pharmacology and Neuroscience, University of North Texas Health Science Center, Fort Worth, TX USA; 5 Texas College of Osteopathic Medicine, University of North Texas Health Science Center, Fort Worth, TX USA

**Keywords:** Hypertension, Early-Life stress, Inflammation, Sex differences, Brain, Kidneys

## Abstract

**Background:**

Poverty, a low resource state, is a common adverse childhood experience (ACE) and early life stress (ELS). People who experienced childhood poverty are at greater risk for developing hypertension during adulthood, with sex differences. To determine the possible mechanisms of these sex differences, we investigated the alterations in blood pressure (BP) and inflammation in the brain and kidneys of rats exposed to an impoverished environment during early life by using the limited bedding and nesting (LBN) rodent model.

**Methods:**

The LBN model mimics childhood poverty by creating a low resource environment on postnatal days 2–9. After weaning, offspring were separated by sex and LBN exposure and were evaluated at 16–18 weeks of age (Adulthood).

**Results:**

LBN males displayed an increase in BP compared to the control (CON), whereas LBN females showed no change. Pro-inflammatory cytokines, IL-17 and TNF-α, were decreased in the brains of LBN vs. CON males, with no alterations in females.

**Conclusion:**

Adult LBN males have elevated BP, while LBN females appear to be protected from increased BP. The reduction in IL-17 and TNF-α in LBN males may serve as a compensatory mechanism to lower BP. This study provides insights into sex differences in BP for adults who experienced childhood poverty.

## Abstract (plain language summary)

People who experience early life stress (ELS), or adverse childhood experiences (ACEs), have an increased risk of developing high blood pressure (BP), cardiovascular, cerebral, and renal diseases as adults with sex differences. To explore these sex differences, we examined the changes in BP and inflammatory factors in the brain and kidney of rats that were exposed to ELS. To induce ELS, we used the limited bedding and nesting (LBN) model. This is a low resource model that reduces bedding and nesting material, during weaning, to create chronic stress for the dam and pups. The LBN model mimics childhood poverty, which is a state of resource deprivation. Poverty is an ACE that affects ~ 333 million children worldwide. In this study, we found that ELS induced high BP, and decreased pro-inflammatory cytokines in the brain and kidneys of LBN males. However, LBN females displayed no changes in brain and kidney pro-inflammatory cytokines and BP. In summary, these sex differences provide context to the development of high BP caused by resource deprivation during early life. Scientists and medical providers should consider sex and organ-specific immunotherapies to assist in lowering BP, mitigating organ damage, and lowering the risk of developing cardiovascular, cerebral, and renal diseases in adults who experienced ACEs, such as poverty.

## Highlights

- Childhood poverty is a major public health concern that increases the risk of hypertension later in life, with sex differences.
- The LBN model induces chronic stress by resource deprivation in early life. This rodent model severely limits the bedding and nesting material during weaning to elucidate the sexually dimorphic mechanisms connecting childhood poverty to hypertension.
- LBN exposure increases BP and decreases pro-inflammatory cytokines in the brain and kidneys in males, but not females. The reduction in localized pro-inflammatory cytokines in LBN males may be a compensatory mechanism to lower BP and/or prevent tissue damage.
- This study helps uncover potential therapeutic targets and bring awareness to the sex differences involved in hypertension during adulthood, initiated by childhood poverty.

## Background

Poverty impacts ~ 333 million children worldwide and increases the risk of developing hypertension, cardiovascular, and cerebrovascular diseases later in life [1]. Poverty is defined as resource deprivation that prevents individuals from maintaining a minimum standard of living [2, 3]. Often, the definition of poverty is limited to its characterization of one’s economic status; an individual making an income less than $30,000 a year in the United States (US) is considered below the poverty line [4]. However, poverty is more than just a lack of income. Poverty restricts access to vital resources, such as food, water, shelter, transportation, education, jobs, safety, and healthcare [5].

Poverty is a major public health concern that disproportionally affects children and is considered an adverse childhood experience (ACE). ACEs are traumatic events that occur before the age of 18 years, which can include neglect, abuse, household dysfunction, and importantly poverty [6–9]. According to the Centers for Disease Control (CDC), approximately 64% of the US population has experienced at least one type of ACE, while 17% reported experiencing four or more ACEs. ACEs can negatively impact a person’s mental and physical health throughout life [10–12].

Epidemiological studies of ACEs and animal models of early life stress (ELS) report an increase in cardiovascular and cerebrovascular diseases during adulthood [9, 11, 13–18]. Studies not only show increased health risks during adulthood, but also sex differences in blood pressure (BP) [19–21]. Multiple studies show that males, especially Black males, have a greater risk of developing hypertension, being diagnosed with hypertension at an earlier time point in adulthood, and display a larger increase in BP compared to females that have experienced ACEs [20, 22–24]. However, these findings are controversial. In more recent studies, adult women display a greater increase in hypertension and other cardiovascular diseases (CVDs) when exposed to ACEs, such as physical and sexual abuse [11, 24–29]. In a new study, by Spikes et al*.* 2025, it was revealed that Black women reported more ACEs and had a stronger positive correlation between childhood trauma and impaired arterial compliance, thus making them more susceptible to CVDs later in life [11, 24]. Therefore, based on these later studies, it can be inferred that maybe women, exposed to ACEs, are at a greater risk of CVDs during adulthood [11, 24–29]. Conversely, some studies suggest no sex differences, while others show that post-menopausal women have equal or greater rates of hypertension compared to males who experienced ACEs [30]. Thus, the notion as to which sex has greater negative health outcomes after being exposed to ACEs is undetermined. These discrepancies in the literature may be attributed to differences in demographic populations, the type and intensity of different ACE exposures, self-reporting in males vs. females, and which cardiovascular output was measured, such as hypertension, stroke, myocardial infarction, and/or cardiac death. From a physiological perspective, these sex differences may also be explained by changes in hormones, neuroendocrine activation, immunological alterations, and autonomic activity [13, 24, 31–33]. Despite the plethora of studies linking ACEs to CVDs during adulthood, the exact mechanisms that predispose this vulnerable population of people to hypertension are unknown and the focus of this study.

Several factors are known to alter BP in humans and rodents exposed to ELS. In this study, we chose to focus on alterations in inflammation. Whereas both circulating and tissue inflammation can play a role in elevating BP [17, 34, 36, 37]. Specifically, an increase in brain and kidney pro-inflammatory cytokines, such as interleukin-17 (IL-17) and tumor necrosis factor-alpha (TNF-α), are shown to increase BP in rodents with or without ELS, with sex differences [37–40]. IL-17 and TNF-α are known to regulate BP, via an increase in systemic and/or tissue inflammation. Elevated renal IL-17 and TNF-α concentrations can alter sodium transporters, increase the production of renin–angiotensin–aldosterone system components, change renal hemodynamics, and cause renal dysfunction to increase BP [36, 41]. These same cytokines in the brain can contribute to cerebrovascular dysfunction and increased blood–brain-barrier (BBB) permeability. An increase in BBB permeability permits the influx of these cytokines into the brain, leading to further damage and inflammation, all of which will increase BP [42]. Additionally, IL-17 and TNF-α can elicit the production of other cytokines, thus generating a pro-inflammatory cytokine storm [43, 44]. Studies from our lab and others have implicated that these cytokines participate in the pathology and pathogenesis of hypertension, ischemic strokes, preeclampsia, hypertension in postpartum preeclamptic women, and importantly, in human and animals exposed to ELS/ACEs [25, 38, 45–48]. Therefore, examining the modifications in and renal and brain inflammation may provide clues into the sex differences associated with CVD risk in people exposed to ACEs.

To study ELS, in the context of a low resource environment, we utilized the limited bedding and nesting (LBN) rodent model, to assess the BP and alterations in inflammation within different regions of the brain and kidney. The LBN model mimics childhood poverty by reducing bedding and nesting material during weaning [45, 49–51]. This model creates a chronically stressed environment, characterized by unpredictable maternal care [45, 49–52]. These fragmented behaviors observed in dams are also observed in people living in impoverished environments [53]. In rodents, the radical changes in maternal care, such as alterations in duration and frequency of grooming, nest building, and rough handling, can mirror neglect and abuse experienced by children during poverty [45, 53]. This ELS model has demonstrated that LBN rodents have alterations in brain structures (such as the hippocampus, amygdala, and prefrontal cortex), hypothalamic–pituitary–adrenal (HPA) axis, cognitive decline, and depression [17, 49, 51]. Despite these novel findings, no studies have investigated sex differences and effect of LBN exposure on BP and inflammation in rodents during adulthood. These studies are necessary because ACEs are common in adults, and the physiological mechanisms connecting ACEs and increased risk of CVDs and cerebrovascular dysfunction are unknown [17, 54].

The objective of this study is to characterize changes in BP and inflammation in the brain and kidney of male and female rats that experienced a low resource (LBN) environment during weaning. We hypothesize that LBN male rats will display elevated BP and inflammation in the brain and kidney, whereas LBN females will display no changes in these measurements. The results from this study will provide insights into the sexually dimorphic mechanisms that link childhood poverty to the development of hypertension during adulthood.

## Methods

### Experimental procedure

Timed-pregnant Sprague–Dawley rats (ENVIGO; Indianapolis, IN) were received on gestational day (GD) 11–12 and were divided into two groups: Control (CON; n = 6) and LBN (n = 7) dams. The rats were housed in a 12-h light/dark cycle with controlled temperature and humidity. Food and water were provided ad libitum throughout the entire experimental protocol. All animals and procedures used in this study were approved by the University of North Texas Health Fort Worth Institutional Animal Care and Use Committee (IACUC) and in accordance with the National Institute of Health (NIH) Guide for the Care and Use of Laboratory Animals.

At GD 20–22, both CON and LBN dams gave birth naturally. From postnatal day (PND) 1–21, the CON dams weaned their pups in a normal environment with the typical amounts of bedding and nesting material. From PND 2–9, LBN dams and pups were relocated to a modified LBN environment, with 75–80% less bedding material, to induce ELS [45, 51, 52]. The LBN environment included a slightly elevated mesh floor on the bottom of a clean cage to allow for droppings to fall through and to provide another layer of stress. During PND 2–6, dam behaviors were recorded, analyzed, and evaluated to calculate entropy, which is a measurement of fragmented behaviors for the dams [49, 55]. The dams were monitored for frequency, total duration, and the mean duration of self-grooming, pup grooming, nursing, nest building, eating and drinking, and transportation of pups in the cage. The measurements were then used to generate an entropy score for our dams, using a computational algorithm [49, 55]. The entropy score utilizes the conditional probability of each behavior, in sequence with other behavior types, to determine if the dams’ behavior was fragmented and unpredictable. Many other research groups have used the entropy score to validate the efficacy of the LBN model in generating ELS [45, 49, 52]. The wire mesh was removed, and normal quantities of bedding and nesting materials were provided to the LBN group on PND 10, which continued until PND 21. After the weaning period, all pups were separated by sex and treatment, then aged to 16–18 weeks of age, which is equivalent to young adulthood in humans [56].

To measure the mean arterial pressure (MAP) carotid catheterization surgery was performed at 16–18 weeks of age. After the BP was recorded, we humanely euthanized the animals to collect blood and organs. The brain and kidney samples were snap-frozen and stored in a −80 °C freezer. Later, the brains were sectioned into cerebrum, brainstem, and cerebellum, whereas the kidneys were separated into medulla and cortex for experimentation and analysis. The organ sections were then homogenized and centrifuged at 10,000G for 20 min to obtain supernatants for future use in colorimetric enzyme-linked immunosorbent assays (ELISAs), namely IL-17 and TNF-α.

### Carotid catheterization surgery for MAP Measurements

At 16–18 weeks of age, we performed carotid catheterization surgeries on all offspring, as described previously by us and others [45, 47, 48]. Briefly, to perform the carotid catheterization surgery, we made an incision into the ventral neck under 2–4% nasal isoflurane, isolated the carotid artery, then implanted a small catheter. We secured the catheter to the artery with surgical thread and weaved it through the dorsal neck. While under anesthesia, core temperature was maintained via a heating pad. This surgery lasted ~ 15 to 20 min per rat. Post-surgery, the rat was placed in a warm, clean cage with a 2 mg tablet of bacon-flavored Rimadyl, to monitor for safe recovery. BP was recorded the next day. Note that MAP was recorded via a PowerLab 16/35 AD Instruments apparatus (ADInstruments, Australia) as performed and described previously [57].

### Inflammation assays

To determine IL-17 and TNF-α concentrations in the brain and kidney, we performed the following ELISAs: IL-17 (DY8410) and TNF-α (DY510-05; R&D Systems, Minneapolis, MN) according to the manufacturer’s instructions and previous studies [47]. Before loading the samples into the 96-well plates, we diluted the kidney cortices to 1:10, while the kidney medullas and brain sections were pipetted without dilution. Both assays were read at 450 nm using the BioTek Epoch 2 microplate reader (Agilent Technologies, Santa Clara, CA) alongside Generation 5 software (Santa Clara, CA).

### Statistics

To analyze dam behavior and entropy score, we utilized the Student’s t-test. Body weight, organ weight, BP, and inflammation were analyzed by a two-way ANOVA with a Fisher’s least significant difference (LSD) post-hoc, using GraphPad Prism 10 (v. 10.0.3; Santa Clara, CA) and Excel (Microsoft). These data were reported as mean ± standard error of the mean (SEM), where statistical significance was defined as *P < 0.05.

## Results

### LBN Induced behavioral changes and increased entropy score in dams

Dams exposed to the LBN model during PND 2–9 exhibited several behavioral modifications in frequency of events, total duration of events, and average time of each event during exposure to the LBN (Table 1). We observed increased frequency of licking and grooming of pups (10.2 ± 1.16 vs. 5.5 ± 1.07A.U.; P = 0.03), self-grooming (7.74 ± 0.68 vs. 2.75 ± 0.64A.U.; P = 0.0009), nest building (3.86 ± 0.84 vs. 1.9 ± 0.33A.U.; P = 0.13), eating (4.23 ± 0.83 vs. 1.65 ± 0.60A.U.; P = 0.06), and off-nest (11.23 ± 1.14 vs. 5.5 ± 0.95 A.U.; P = 0.008). Furthermore, the total duration of licking and grooming the pups (441.65 ± 52.94 vs. 283.80 ± 40.89 s; P = 0.07), self-grooming (258.83 ± 22.61 vs. 98.69 ± 30.34 s; P = 0.002), and eating (373.80 ± 80.49 vs. 137.73 ± 32.42 s; P = 0.06) also increased. However, there were no significant changes in total duration of nest building (144.11 ± 42.81 vs. 114.37 ± 29.20 s; ns) and off-nest time (571.33 ± 150.59 vs. 405.15 ± 178.83 s; ns). There was a significant decrease in total duration of nursing time (1,803.63 ± 150.85 vs. 2554.70 ± 166.52 s; P = 0.01) with LBN dams, but no change in nursing event frequency (9.89 ± 1.57 vs. 7.65 ± 0.82A.U.; ns). Overall, no significant changes in the average time of each behavior were observed during this period (Table 1). To validate the exposure of chronic stress on the dams and pups, an entropy score was calculated. The LBN dams showed increased entropy (1.16 ± 0.05 vs. 0.83 ± 0.02A.U.; P = 0.001) compared to the CON. Other groups have also demonstrated a higher entropy score in LBN dams, reflecting a chronically stressed environment with fragmented maternal behaviors (Table 1) [45, 51, 52].

**Table 1:** Behavioral Changes of Postpartum Dams (PND 2–6) Exposed to LBN

Measures	Number of events			Total duration (s)			Mean duration of each event (s)		
**Behaviors**	**CON (Mean ± SEM)**	**LBN (Mean ± SEM)**	**P-Value**	**CON (Mean ± SEM)**	**LBN (Mean ± SEM)**	**P-Value**	**CON (Mean ± SEM)**	**LBN (Mean ± SEM)**	**P-Value**
**Licking & Grooming (LG)**	5.5 ± 1.07	10.2 ± 1.16	**0.03**	283.80 ± 40.89	441.65 ± 52.92	0.07	64.15 ± 14.63	47.76 ± 2.50	0.17
**Self-Grooming (SG)**	2.75 ± 0.64	7.74 ± 0.68	**0.0009**	98.69 ± 30.34	258.83 ± 22.61	**0.002**	22.23 ± 2.71	32.15 ± 3.78	0.1
**Nursing (N)**	7.65 ± 0.82	9.89 ± 1.57	0.34	2554.70 ± 166.52	1803.63 ± 150.85	**0.01**	485.69 ± 80.81	317.28 ± 70.71	0.17
**Nest-Building (NB)**	1.9 ± 0.33	3.86 ± 0.84	0.13	114.37 ± 29.20	144.11 ± 42.81	0.64	31.43 ± 6.92	29.65 ± 6.07	0.86
**Eating (E)**	1.65 ± 0.60	4.23 ± 0.83	0.06	137.73 ± 32.42	373.80 ± 80.49	0.06	53.74 ± 11.95	67.39 ± 11.12	0.45
**Carrying Pups (C)**	0.3 ± 0.24	0.69 ± 0.16	0.19	4.91 ± 4.54	6.33 ± 1.87	0.74	2.64 ± 2.28	3.84 ± 1.07	0.6
**Off-Nest (O) = O + M**	5.5 ± 0.95	11.23 ± 1.14	**ss0.008**	405.15 ± 178.83	571.33 ± 150.59	0.51	128.31 ± 48.92	97.53 ± 34.14	0.61
	**CON (Mean ± SEM)**			**LBN (Mean ± SEM)**			**P-Value**		
**Entropy**	0.83 ± 0.02			1.16 ± 0.05			**0.001**		

All data for the behavior observations were analyzed with two-way ANOVA and the Fisher’s LSD test for *post-hoc* test for comparisons. Entropy analysis was compared by using student t-test. Comparisons with P < 0.05 are bolded. Comparisons with 0.050 < P < 0.100 are underlined. Both significant and marginal comparisons are posted

### Body weight and organ weight analysis

Body weight, brain weight, total kidney weight, and heart weight of adult offspring showed no differences between treatment in male or female LBN vs. CON rats (Table 2). However, there was a sex effect, with males having a larger body weight and smaller brain-to-body weight ratio than females in both LBN and CON rats (Table 2).

**Table 2:** Measurement of Body Weight, and Organ Weights

	CON M	LBN M	CON F	LBN F	Treatment effect (P-Value)	Sex effect (P-Value)	Interaction (P-Value)
	(AVG ± SEM)	(AVG ± SEM)	(AVG ± SEM)	(AVG ± SEM)			
Body Weight (g)	446.13 ± 13.28	444.43 ± 12.01	256.00 ± 6.71	259.25 ± 5.92	> 0.9999	< 0.0001	0.7369
Brain Weight (g/kg of BW)	4.45 ± 0.12	4.44 ± 0.07	7.18 ± 0.17	6.90 ± 0.18	0.2933	< 0.0001	0.3902
Total Kidney Weight (g/kg of BW)	5.64 ± 0.09	5.73 ± 0.14	5.81 ± 0.14	5.66 ± 0.19	0.8785	0.7696	0.3981
Heart Weight (g/kg of BW)	3.00 ± 0.09	3.21 ± 0.13	3.24 ± 0.19	3.25 ± 0.23	0.6408	0.2100	0.6785

All measurements at 16–18 weeks (adulthood) were analyzed with two-way ANOVA and the Fisher’s LSD test for *post-hoc* test for comparisons. Comparisons with P < 0.05 are bolded

### LBN & sex: effects on BP, HR, and autonomic activity

**Fig. 1:**
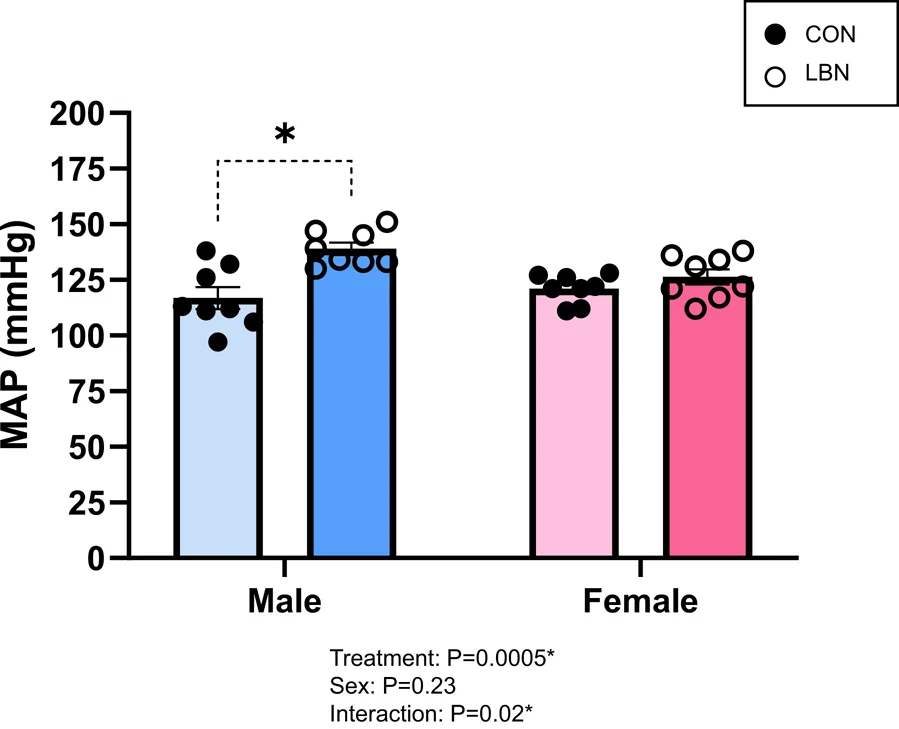
**A**) Systolic BP, **B**) diastolic BP, **C**) MAP, and **D**) HR were measured in LBN males (*n* = 7–8), CON males (*n* = 7–8), LBN females (*n* = 8), and CON females (*n* = 8). All bar graphs display individual data points with each group’s mean ± SEM, with significance determined at *P* < 0.05. Groups were statistically analyzed with two-way ANOVA and the Fisher’s LSD test for *post-hoc* analysis. Individual data points are represented by closed circles for CON offspring and open circles for LBN offspring. Male bar graphs are blue and female bar graphs are pink

**Fig. 2:**
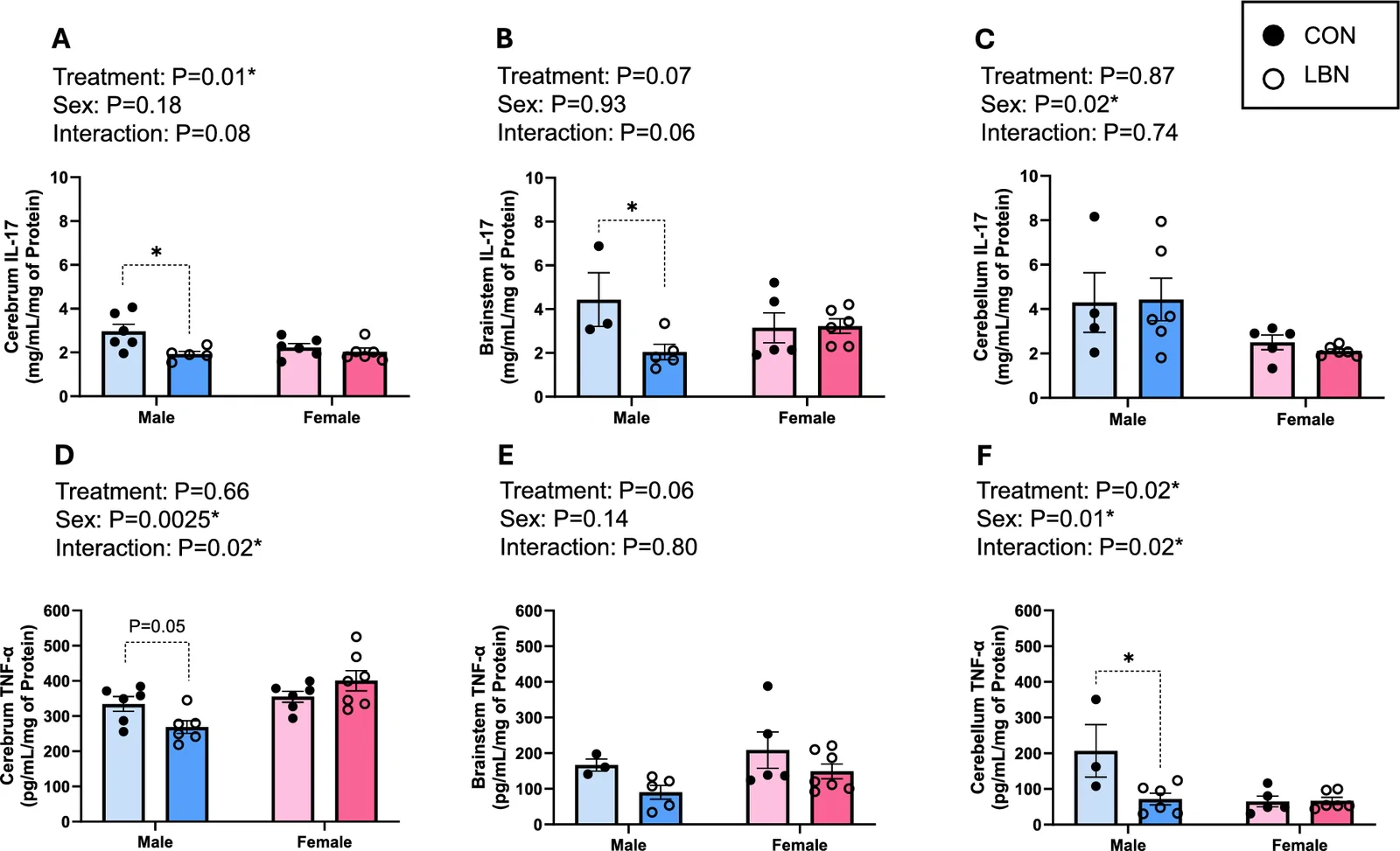
**A**) Cerebrum, **B**) brainstem, and **C**) cerebellum IL-17 concentrations were measured in LBN males (n = 5–6), CON males (n = 3–6), LBN females (n = 5–6), and CON females (n = 6). **D**) Cerebrum, **E**) brainstem, and **F**) cerebellum TNF-α concentrations were measured in LBN males (n = 5–6), CON males (n = 3–6), LBN females (n = 6–7), and CON females (n = 5–6). All bar graphs display individual data points with each group’s mean ± SEM, with significance determined at *P* < 0.05. Groups were compared with two-way ANOVA and the Fisher’s LSD test for *post-hoc* analysis. Individual data points are represented by closed circles for CON offspring and open circles for LBN offspring. Male bar graphs are blue and female bar graphs are pink

MAP was measured in adult CON and LBN rats in adulthood (Figs. 1A-D and 2A-B). MAP showed both a treatment and interaction effect, in which LBN exposure had an effect in males but no effect in females (Fig. 1C). In fact, LBN males exhibited a 16% (~ 23 mmHg) increase in MAP (139 ± 3 vs. 117 ± 5 mmHg; P = 0.0001) compared to the CON males (Fig. 1C). There were no changes in MAP between LBN and CON females.

### LBN & sex: effects on brain inflammation

IL-17 and TNF-α were examined in different segments of the brain: cerebrum, brainstem, and cerebellum outlined in figs. 2A-F. There was a trend in the interaction effect (Sex X LBN), observed in the cerebrum (P = 0.08) and brainstem (P = 0.06) for IL-17. There were no significant changes in cerebral IL-17 in females (Fig. 2A). Although in figs. 2A-B, LBN males exhibited a decrease in IL-17 concentrations in the cerebrum (1.93 ± 0.13 vs. 2.96 ± 0.34 mg/mL/mg of Protein, P = 0.01) and brainstem (2.04 ± 0.35 vs. 4.43 ± 4.43 ± 1.23 mg/mL/mg of Protein; P = 0.02). There was no difference in cerebellar IL-17 between LBN vs. CON rats within each sex, but there was an overall sex difference (P = 0.02) with females having less IL-17 compared to males (Fig. 2C).

Cerebral TNF-α concentration was decreased for LBN vs. CON males (268.81 ± 17.99 vs. 334.28 ± 20.91 pg/mL/mg of Protein; P = 0.05), with no differences in LBN vs. CON females (Fig. 2D). No differences were observed with brainstem TNF-α (Fig. 2E). However, cerebellum TNF-α showed a fourfold decrease between LBN vs. CON males (71.90 ± 16.43 vs. 206.79 ± 73.65 pg/mL/mg of Protein; P = 0.003), with no changes presented in females (Fig. 2F).

### LBN & sex: effects on kidney inflammation

**Fig. 3:**
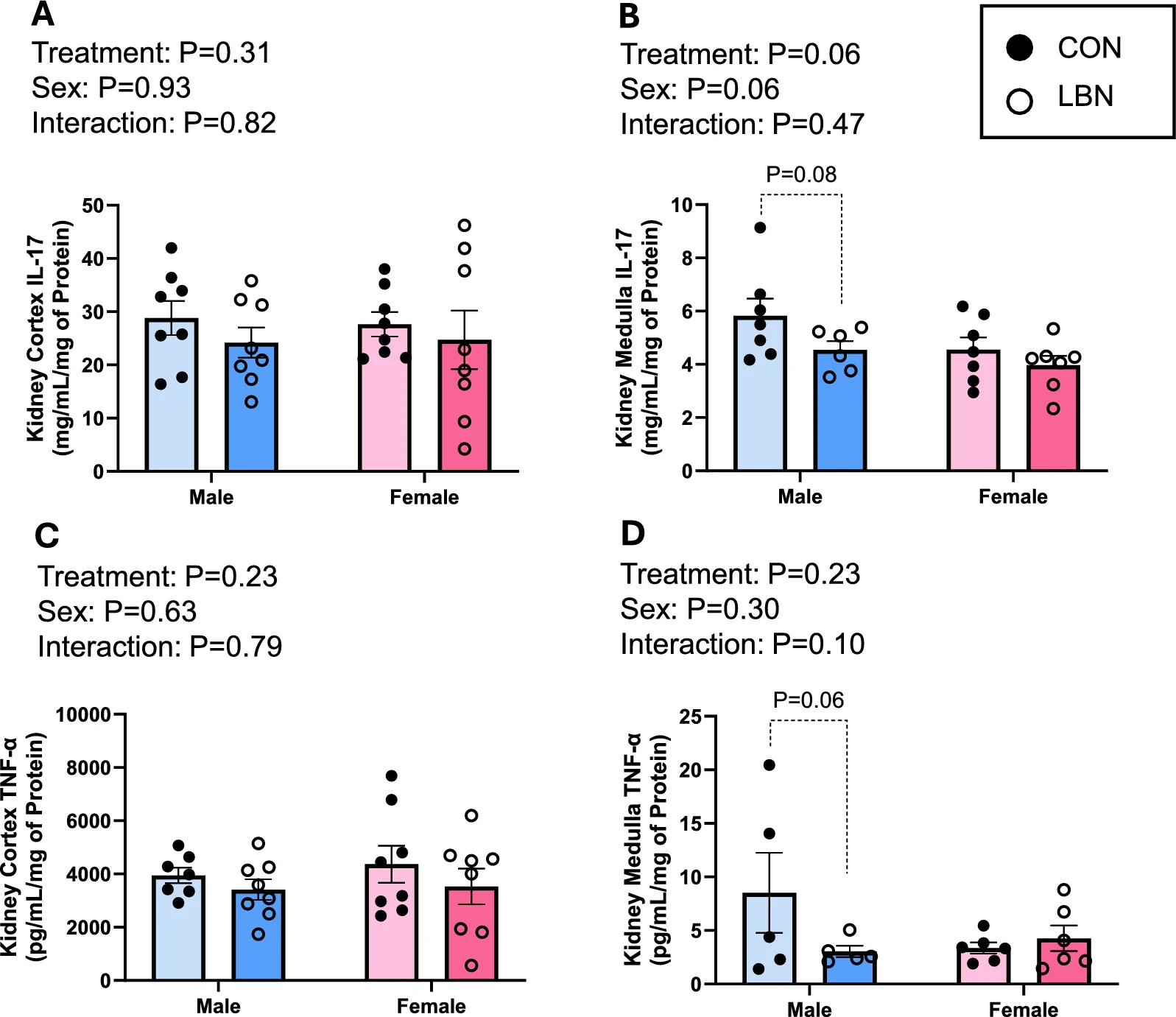
**A**) Kidney cortex and **B**) kidney medulla IL-17 concentrations were measured in LBN males (n = 6–8), CON males (n = 7–8), LBN females (n = 7–8), and CON females (n = 7–8). **C**) Kidney cortex and **D**) medulla TNF- α concentrations were also measured in LBN males (n = 5–8), CON males (n = 5–7), LBN females (n = 6–8), and CON females (n = 6–8). All bar graphs display individual data points with each group’s mean ± SEM, with significance determined at *P* < 0.05. Groups were compared with two-way ANOVA and the Fisher’s LSD test for *post-hoc* analysis. Individual data points are represented by closed circles for CON offspring and open circles for LBN offspring. Male bar graphs are blue and female bar graphs are pink

Concentrations of IL-17 and TNF-α were also investigated within the kidney cortex (Figs. 3A & C) and medulla (Figs. 3B & D). IL-17 within the kidney cortex revealed no effects with LBN exposure and/or sex (Fig. 3A). However, IL-17 in the kidney medulla tended to decrease in LBN vs. CON males (4.55 ± 0.33 vs. 5.83 ± 0.64 mg/mL/mg of Protein; P = 0.08) (Fig. 3B). No differences exist between sex and LBN exposure vs. CON females for kidney medulla IL-17.

In the kidney cortex, there was no differences in LBN exposure and/or sex between groups for TNF-α (Fig. 3C). Although, kidney medulla TNF-α tended to decrease in LBN vs. CON males (3.04 ± 0.53 vs. 8.52 ± 3.74 pg/mL/mg of Protein; P = 0.06) (Fig. 3D).

## Discussion

**Fig. 4:**
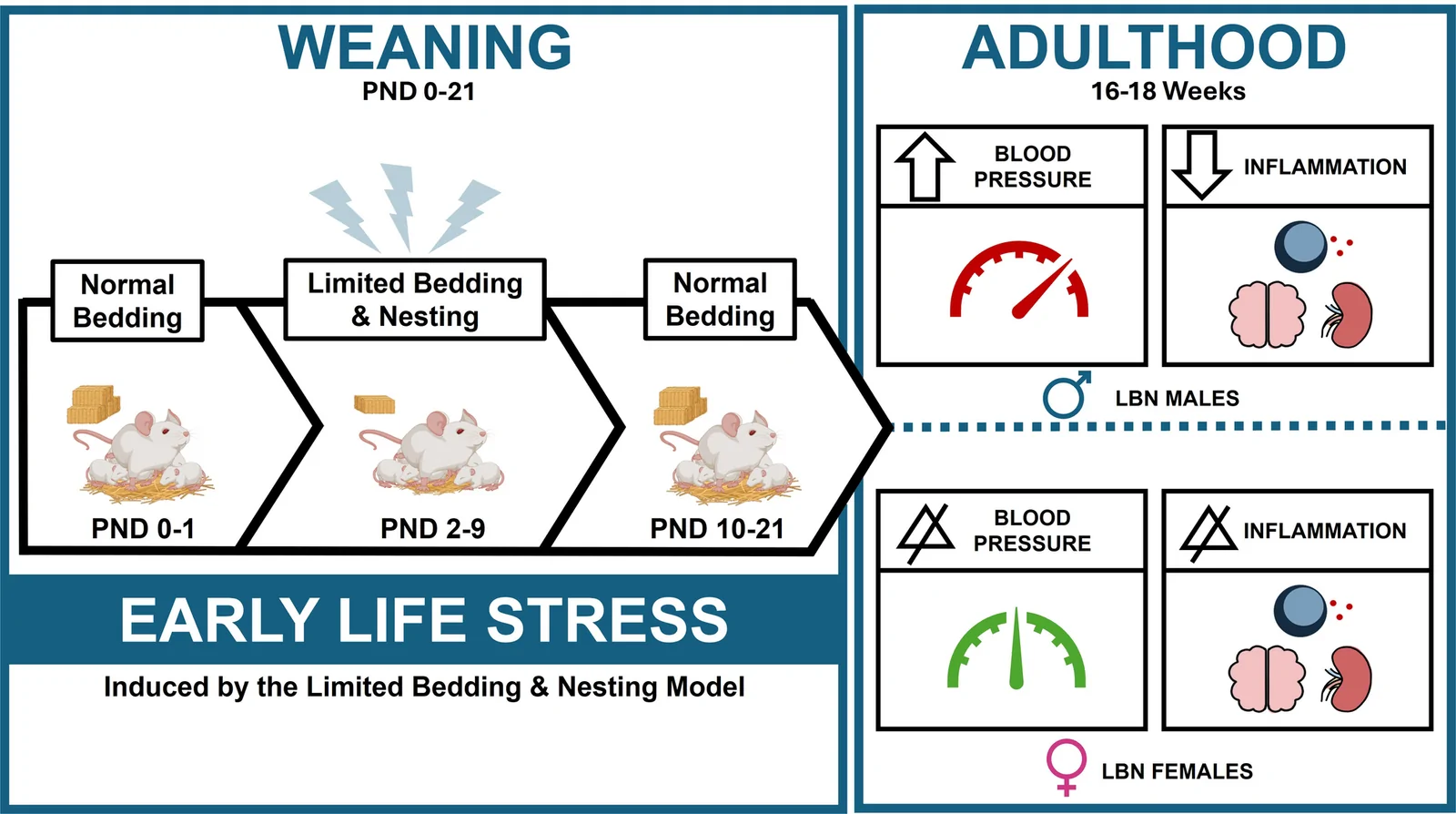
A Graphic design summarizing the major findings of this study

LBN exposure induces sex differences in BP and inflammation in the brain and kidney (Fig. 4). Specifically, LBN males exhibit an increase in BP and decreased pro-inflammatory markers in the cerebrum, brainstem, and cerebellum. LBN females depicted no differences in BP and no alterations in brain or kidney inflammation. The sex differences in inflammation may be a compensatory response to BP, in which LBN males show a reduction pro-inflammatory cytokines to mitigate brain damage from an increase in BP. Meanwhile, LBN females do not show changes in inflammation, since BP is not different between groups. It is important to note that these sexually dimorphic differences in BP and brain/kidney inflammation may also be explained by differences in sex hormones, which was not examined in this study [11, 19–21, 25, 29].

Children exposed to ACEs, including poverty, have an increased risk of poor health outcomes such as a heightened risk of hypertension, inflammation, and stroke as adults [9, 11, 13–18, 20, 22, 23, 25, 27–30, 60]. Several clinical studies show that ACEs are linked to sex differences in the presentation of hypertension [8, 11, 14, 20, 24, 25, 27–30, 32, 61–63]. For example, in Huang et al*.’s* study, they found that persons experiencing ACEs increased the risk of hypertension later in life, indirectly, through cardiometabolic dysregulations (such as hyperlipidemia and hyperglycemia), systemic inflammation, and obesity [61]. In a similar study, conducted in the United Kingdom, Deschenes et al*.* found that British civil service employees, who reported ACEs had a higher risk for developing heart coronary disease, partially mediated through cardiometabolic dysregulation and hypertension [32]. Additionally, Su et al*.’s* 2015 longitudinal study demonstrated a strong, positive relationship between the number of ACES and BP; participants who were exposed to more ACEs exhibited a larger increase in BP during early adulthood compared to those who did not experience any ACEs [8].

The association between elevated BP and ACEs is not only observed in clinical studies, but also in animal models of ELS, using maternal separation and/or early weaning models [64–66]. Franco et al*.’s* 2013 study revealed that 25-week-old adult Wistar male rats exposed to early weaning were hypertensive with extensive oxidative stress [64]. Reho and Fisher 2015 investigated changes in vascular function and BP in a maternal separation rodent model. In these studies, they found that maternal separation caused an increase in arterial contractility at PND 21, but no changes in BP. However, at PND 35, the relationship was reversed, in which BP was increased while arterial contractility was unchanged [65]. Genest et al*.* 2004 showed that neonatal maternal separation led to a stark 20% increase in MAP in males, but no changes in females at 8–10 weeks of age [66]. Note that these results are like our findings, in which LBN males, during adulthood, had significantly increased MAP, while females remained unchanged. Together, these data show that ELS, whether it be via maternal separation or our model of LBN, is linked to hypertension.

The LBN model reduces resources to induce chronic stress in dams and pups during weaning. Typically, the LBN model is used to evaluate outcomes of depression and cognitive dysfunction along with changes in brain morphology [49, 52, 67, 68]. However, our study utilized this model to investigate the mechanisms that link living in a low resource environment during childhood to changes in BP regulation and organ inflammation. To validate the LBN model, we recorded and analyzed the dams’ behavior during LBN exposure. We found changes in licking and grooming, self-grooming, nursing, and time off-nest, which correlated with changes that others have observed with this model [49, 69]. Moreover, we calculated an entropy score, which measures maternal behavior fragmentation, and showed that our score was higher than the CONs, consistent with findings from others who use this model have found [49] **(**Table 1**)**.

We observed sex differences in adult BP, with LBN males showing an increase and LBN females displaying no changes. Many potential mechanisms could facilitate these differences in BP between males and females, which include alterations in autonomic activity, inflammation, oxidative stress, vascular dysfunction, HPA axis dysfunction, endothelin-1, and decreased NO bioavailability. However, in this study, we chose to focus on inflammation in the kidney and brain using the LBN model of ELS, due to to its crucial role in BP regulation.

Two important pro-inflammatory cytokines that we investigated in the brains and kidneys are IL-17 and TNF-α, which both directly and indirectly influence BP regulation and can cause hypertension [38, 80–83]. In this study, we hypothesized that both IL-17 and TNF-α would be increased in the brain and kidneys of hypertensive LBN males and not changed in normotensive LBN females. While our hypothesis was accurate with LBN females, the LBN males displayed the opposite of what we predicted. LBN males showed a reduction in pro-inflammatory cytokines (IL-17 and TNF-α) despite having elevated BP. Therefore, the decrease in brain and kidney pro-inflammatory cytokines may be compensatory. This compensatory relationship observed in this study is not uncommon, as others have observed these same changes in pro-inflammatory markers in the presence or hypertension [87]. Thus, the changes in organ inflammation in LBN rodents may be an attempt to lower and/or maintain normal BP and to prevent tissue damage. The literature supports these findings, in which the relationship between BP and inflammation can be complex. Whereas, most studies show a positive correlation between BP and inflammation, especially in individuals or animals with hypertension [86]. However, some studies have demonstrated a U-shaped relationship with BP and inflammation, where extended states of untreated and chronic hypertension are associated with decreases in inflammation [87].

### Limitations and future directions

It is important to note that this study is observational and not causal. Although the findings in this study are novel, we cannot reveal the exact timing, order of sequence of physiological events, and/or how each physiological change influences the other changes, i.e., how changes in inflammation and BP influence each other. To address these issues, longitudinal studies (before puberty, after puberty, and early adulthood) and gain of function and/or inhibition studies with inflammation need to be performed. Other pro-inflammatory (IL-6, IL-1β and C-reactive protein) and anti-inflammatory (IL-4 and IL-10) cytokines should be explored in specific brain regions (such as the rostral ventrolateral medulla, paraventricular nucleus, and sections of the hippocampus), known to change BP. Additionally, it would be helpful to confirm these trends in inflammatory alterations via western blot, immunohistochemistry, and flow cytometry in brain and kidney, which help identify the specific location and cells within the organs that demonstrate these changes.

In addition, this study did not measure the changes in sex hormones in response to BP and brain and kidney inflammation in LBN rats. However, observing sex hormones, such as estrogen, progesterone, and testosterone, and other androgens, in both males and females, may shed light on the sex differences observed in our study.

Sex hormones are known to modulate BP, oxidative stress, and inflammation [31, 95–97]. Several studies show that estrogen and/or estrogen receptor activation, in premenopausal women, reduces the production of reactive oxygen species, lowers inflammation, increases antioxidant capacity, and enhances NO production [31, 34, 98, 99]. Estrogen administered to male rats with ischemic reperfusion injury displayed a reduction in infarct size and cardiac injury, suggesting that estrogen has cardioprotective effects in males too [99]. These studies indicate that estrogen is protective against the development of CVDs in both males and females. Future studies will address the role of sex hormones in the pathogenesis of hypertension in the context of ELS in adult offspring.

### Perspectives and significance

In summary, our data display sex differences, in which adult LBN males have elevated BP and LBN females are nor-motensive, and therefore protected from increased BP elevation.. Furthermore, we detected no changes in pro-inflammatory cytokines in the brain and kidneys of LBN females but found a decrease in LBN males. The reduction in pro-inflammatory cytokines, IL-17 and TNF-α, in LBN males may serve as a compensatory mechanism to lower BP and/or prevent tissue damage. Identifying the sexually dimorphic alterations in the pathology of adults exposed to an ELS may help to derive novel treatments for patients who have experienced ACEs.

### Societal implications and conclusions

Understanding the mechanism of hypertension in animals that experience ELS will help scientists and physicians better understand the pathology and pathogenesis of hypertension in individuals who have experienced poverty as an ACE. Poverty is a pervasive and prevalent issue that continues to impact the health and well-being of many individuals and families worldwide. Unfortunately, with the lag in wage growth and increase in income inequality, along with a rise in inflation, food costs, high unemployment, and lack of affordable housing, it is predicted that childhood poverty rates will increase [115]. To change the negative trajectory of poor health outcomes in people that have experienced ACEs, we advocate that 1) scientists conduct more experiments to determine the impact of resource deprivation (i.e., poverty) on the development of hypertension with sex differences, and 2) the mechanisms that link ELS/ACEs to hypertension development with sex differences.

## Data Availability

The data that support the findings of this study are available from the corresponding author, [Mark Cunningham], upon reasonable request.

## Abbreviations

ACE: Adverse childhood experience
A.U: Arbitrary units
BBB: Blood–brain-barrier
BP: Blood pressure
CVDs: Cardiovascular diseases
CDC: Centers for disease control
ELS: Early life stress
ELISAs: Enzyme-linked immunosorbent assays
GD: Gestational Day
HPA: Hypothalamic–pituitary–adrenal
IACUC: Institutional animal care and use committee
IL-17: Interleukin-17
LSD: Least significant difference
NIH: National institutes of health
PND: Postnatal day
RPM: Revolutions per minute
SEM: Standard error of the mean
TNF-α: Tumor necrosis factor
UNTHFW: University of North Texas Health Fort Worth
