# Correction: Rats exposed to a low resource environment in early life display sex differences in blood pressure, autonomic activity, and brain and kidney pro-inflammatory markers during adulthood

**DOI:** 10.1186/s13293-026-00983-w

**Published:** 2026-10-23

**Authors:** Jonna Smith, Savanna Smith, Kylie Jones, Angie Castillo, Jessica L. Bolton, Ahfiya Howard, Luis Colon-Perez, Faith Femi-Ogunyemi, Allison Burkes, Mark Cunningham

**Affiliations:** 1 Department of Physiology and Anatomy, University of North Texas Health Science Center, Fort Worth, TX USA; 2 Neuroscience Institute, Georgia State University, Atlanta, GA USA; 3 School of Social Work, East Texas A&M University, Commerce, TX USA; 4 Department of Pharmacology and Neuroscience, University of North Texas Health Science Center, Fort Worth, TX USA; 5 Texas College of Osteopathic Medicine, University of North Texas Health Science Center, Fort Worth, TX USA

**Correction: Biol Sex Differ 17, 43 (2026)**

**https://doi.org/10.1186/s13293-026-00842-8**

Following publication of the original article [1], the authors identified a data recording error and methodological redundancies in the presented figures. The authors have updated the manuscript to ensure scientific accuracy and clarity. The specific corrections are detailed below:

**1. Corrections and Revisions to Fig. 1**

- **Data Correction**: A recording error was identified in the blood pressure graphs, where the systolic blood pressure (SBP) and diastolic blood pressure (DBP) value of 65 mmHg was incorrectly entered. The graphs have been replotted. The corrected data displays the exact same physiological trends as originally reported.
- **Simplification and Redundancy Removal**: To eliminate redundant data, the SBP and DBP graphs were removed, as both parameters mirrored the trends captured by the mean arterial pressure (MAP) tracings.
- **Methodological Refinement**: The SBP, DBP, and heart rate (HR) graphs were ultimately removed from the manuscript. These parameters were derived indirectly via software automation using MAP tracings, which provides a less accurate measurement of these specific variables and added no additional value to the study.

**2. Removal of Fig. 2 and Associated Spectral Analyses**

- **Methodological Limitations**: Figure 2 (panels A and B) has been removed from the manuscript. These figures utilized low-frequency blood pressure variability (LFBPV) and high-frequency heart rate variability (HFHRV) as proxies for sympathetic nerve activity (SNA) and parasympathetic nerve activity (PNA), respectively.
- **Scientific Rationale**: Spectral analysis of blood pressure and heart rate provides an indirect estimation rather than a definitive, direct measurement of autonomic activity. While utilized within the scientific community, these techniques are indirect, controversial, and do not represent the gold-standard approach. Conclusive verification of autonomic nervous system activity requires direct measurement via microneurography.
- **Confounding Variables**: Rodent physiological measurements of blood pressure and heart rate are highly susceptible to external confounding variables. To avoid the introduction of potential artifacts, Figure 2 has been omitted entirely.

**3. Textual Amendments**

To maintain consistency with the figure omissions, all corresponding text, references, and data interpretations regarding the removed parameters have been deleted from the **Title**, **Methods**, **Results**, and **Discussion** sections of the manuscript.

The incorrect Fig. 1 is given below

**Figure:**
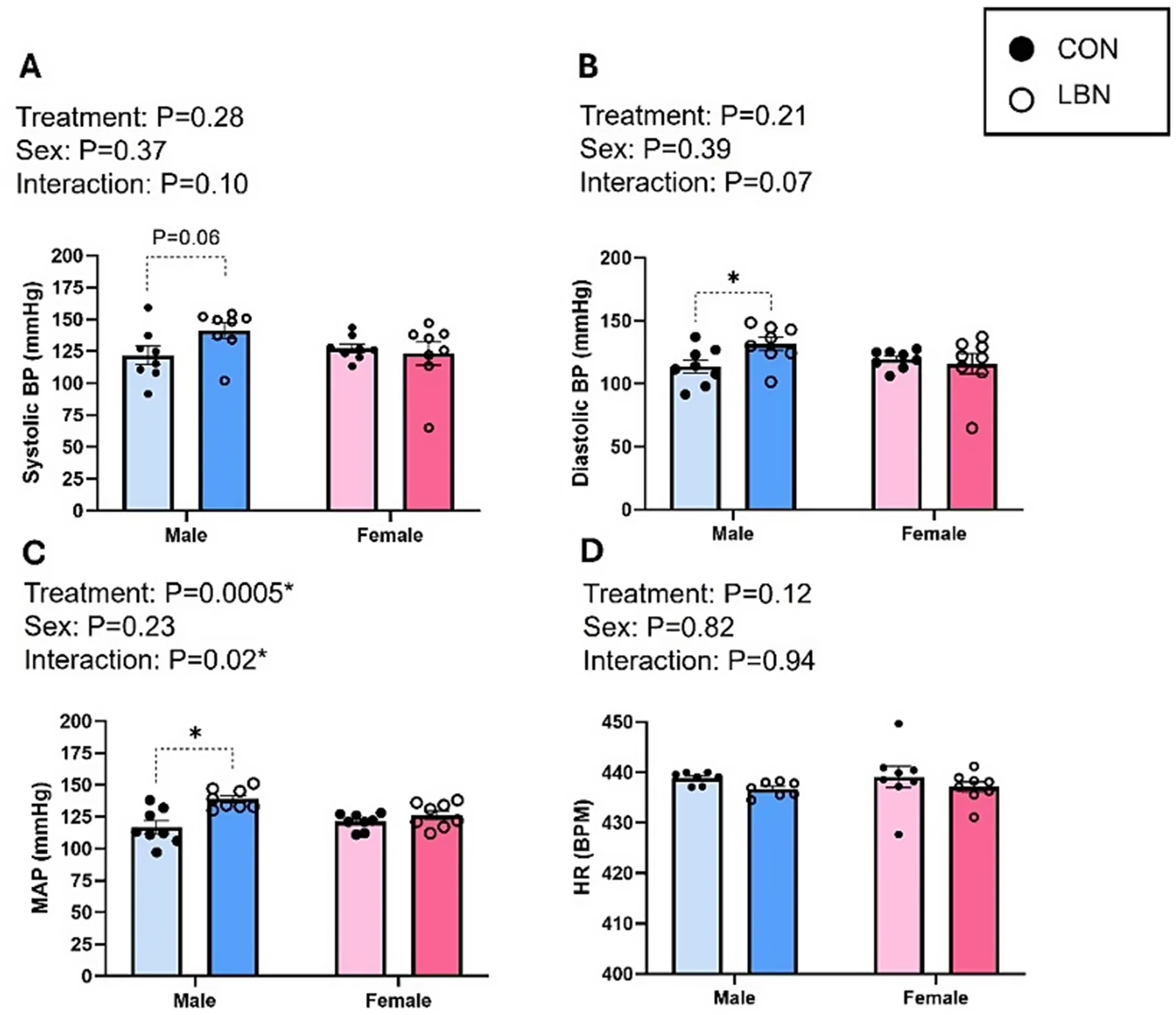

The corrected Fig. 1 is given below 

**Figure:**
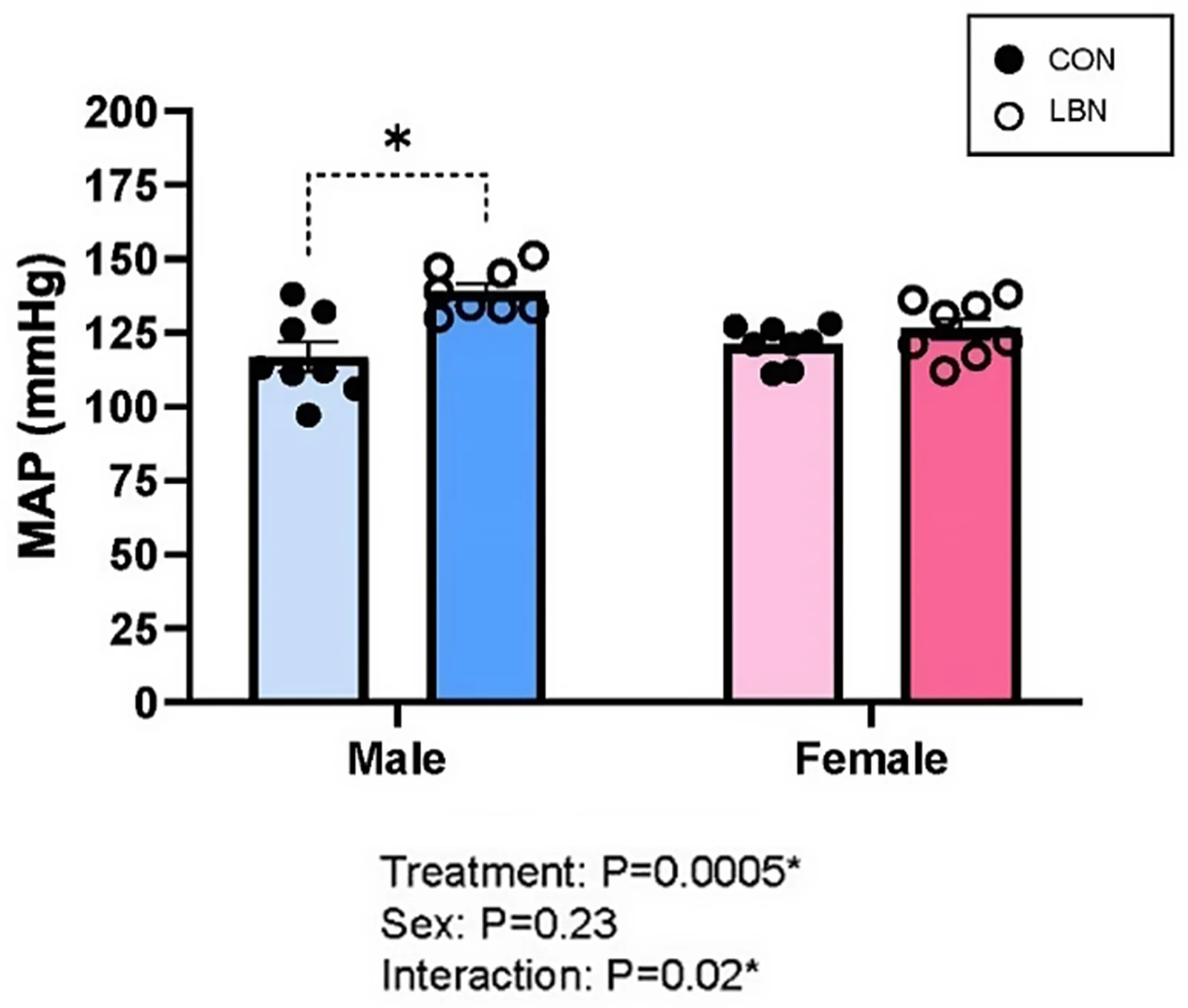

The original article [1] has been corrected. 
